# A subject-independent pattern-based Brain-Computer Interface

**DOI:** 10.3389/fnbeh.2015.00269

**Published:** 2015-10-20

**Authors:** Andreas M. Ray, Ranganatha Sitaram, Mohit Rana, Emanuele Pasqualotto, Korhan Buyukturkoglu, Cuntai Guan, Kai-Keng Ang, Cristián Tejos, Francisco Zamorano, Francisco Aboitiz, Niels Birbaumer, Sergio Ruiz

**Affiliations:** ^1^Institute of Medical Psychology and Behavioral Neurobiology, Medical Faculty, University of TübingenTübingen, Germany; ^2^Institute for Medical and Biological Engineering, Schools of Engineering, Medicine and Biology, Pontificia Universidad Católica de ChileSantiago de Chile, Chile; ^3^Department of Psychiatry and Section of Neuroscience, School of Medicine, Pontificia Universidad Católica de ChileSantiago de Chile, Chile; ^4^Graduate School of Neural and Behavioral Sciences, University of TübingenTübingen, Germany; ^5^Institut de Recherche en Sciences Psychologiques, Université Catholique de LouvainLouvain-la-Neuve, Belgium; ^6^Neural and Biomedical Technology Department, Institute for Infocomm ResearchSingapore, Singapore; ^7^Department of Electrical Engineering and Biomedical Imaging Center, Pontificia Universidad Católica de ChileSantiago, Chile; ^8^División de Neurociencia, Centro de Investigación en Complejidad Social, Facultad de Gobierno, Universidad del DesarrolloSantiago, Chile; ^9^Unidad de Imágenes Avanzadas, Clínica Alemana, Universidad del DesarrolloSantiago, Chile; ^10^Departamento de Psiquiatría, Centro Interdisciplinario de Neurociencia, Escuela de Medicina, Pontificia Universidad Católica de ChileSantiago, Chile; ^11^Ospedale San Camillo, Istituto di Ricovero e Cura a Carattere ScientificoVenezia, Italy

**Keywords:** neurofeedback, BCI, subject-independent classification, emotion imagery, common spatial patterns

## Abstract

While earlier Brain-Computer Interface (BCI) studies have mostly focused on modulating specific brain regions or signals, new developments in pattern classification of brain states are enabling real-time decoding and modulation of an entire functional network. The present study proposes a new method for real-time pattern classification and neurofeedback of brain states from electroencephalographic (EEG) signals. It involves the creation of a fused classification model based on the method of Common Spatial Patterns (CSPs) from data of several healthy individuals. The subject-independent model is then used to classify EEG data in real-time and provide feedback to new individuals. In a series of offline experiments involving training and testing of the classifier with individual data from 27 healthy subjects, a mean classification accuracy of 75.30% was achieved, demonstrating that the classification system at hand can reliably decode two types of imagery used in our experiments, i.e., happy emotional imagery and motor imagery. In a subsequent experiment it is shown that the classifier can be used to provide neurofeedback to new subjects, and that these subjects learn to “match” their brain pattern to that of the fused classification model in a few days of neurofeedback training. This finding can have important implications for future studies on neurofeedback and its clinical applications on neuropsychiatric disorders.

## Introduction

A variety of studies using Brain-Computer Interfaces (BCI) and neurofeedback have demonstrated that individuals can be trained to gain control of different brain signals. Researchers have anticipated that long-term BCI training may lead to neuroplastic changes, potentially opening up new treatment approaches for certain psychiatric disorders, e.g., depressive disorders, schizophrenia, and attention deficit hyperactivity disorder (Strehl et al., 2006; Choi et al., 2011; Linden et al., 2012; Ruiz et al., 2013a,b; Young et al., 2014). Many of these studies are based on the idea that patients can be trained to correct their abnormal brain activation to produce healthy brain activation, aided by the feedback of their own brain activity.

While earlier BCI studies have mostly focused on modulating specific brain regions or signals, new developments in pattern classification of brain states are enabling real-time decoding and modulation of an entire functional network (Cox and Savoy, 2003; Peltier et al., 2009; Hollmann et al., 2011; Laconte, 2011; Sitaram et al., 2011; Rana et al., 2013; Sato et al., 2013; Niazi et al., 2014; Ruiz et al., 2014b). However, a major methodological concern arises from these approaches: the prior studies have focused on building pattern classifiers to decode subject-specific brain patterns, and it is not clear if a general approach could be developed such that a classifier trained on brain signals from a group of individuals could be used to distinguish between any two given specified brain states. Progress in this type of classifier using a fused model, created by combining data from several subjects, hereafter called subject-independent pattern classifier, is increasingly considered to be necessary. The goal is to decode, in real-time, from an individual's brain signals without having to first train the pattern classifier on his subject-specific data (Rana et al., 2013; Ruiz et al., 2014b). One potential application of such a technique involves training neuropsychiatric patients to correct and reverse their abnormal patterns of brain activity: instead of providing feedback of their own brain activity (purportedly abnormal due to the presence of an active neuropsychiatry disorder), future neurofeedback experiments could reinforce patients whenever they are able to emulate patterns of activity similar to those of healthy brains. A system that is capable of providing neurofeedback in this manner has to fulfill two basic requirements: (1) The feature extraction method must consider information from spatial, temporal and spectral domains to be able to encapsulate the entire functional network. (2) The feature extraction method has to be generalizable to multiple subjects in order to be able to construct a fused classification model.

It is to be noted that there have been a few published reports of subject-independent pattern classification, although they have been limited to event related potentials and were not demonstrated to be generalized to other brain states. A previous attempt to create a subject-independent classification model from P300 event related potentials for a BCI spelling application was reported in Lu et al. (2009). More recently, similar results were reported by Jin et al. (2013) and Kindermans et al. (2014). The above studies aimed to build a subject-wide model of ERP signals to minimize calibration time for new users of the BCI speller application. Although the results were promising, the reported methods were limited to event related signals with very specific spatial and temporal characteristics which might not be suitable for a more general application of classifying between any two arbitrary brain states.

Another study by Fazli et al. (2009) used Common Spatial Patterns (CSP) to extract features from the EEG data of event-related synchronization and desynchronization during motor imagery. Multiple linear classifier were combined to form a merged classifier that enabled classification on new data without calibration.

These reports show that group-based, real-time classification on signals with well-known characteristics is possible. However, in a paradigm that reinforces brain-pattern matching by enabling group-based neurofeedback of more general patterns of activity in functional networks, the characteristics of the signal in terms of spatial and spectral distribution are not always know in advance. However, the fact that CSP is a data-dependent method makes it an interesting candidate for such an approach (Krusienski et al., 2012). CSP constructs spatial filters from the data that, when projected, identify important parts of the data, i.e., regions on the scalp that contain information for the discrimination of two classes (see Koles et al., 1990; Koles, 1991; Müller-Gerking et al., 1999; Ramoser et al., 2000; Blankertz et al., 2008). The above-mentioned study by Fazli et al. shows that CSP can be used in a group-based setting, however, the efficacy was only shown in offline tests. An example for the use of the CSP method in a single-trial BCI setting is given in Guger (Guger et al., 2000). Here, motor imagery data is used to train subject-specific classifiers based on CSP, and were also successfully tested in real-time experiments.

Toward our long-term goal of developing a BCI for training neuropsychiatric patients to produce brain-patterns similar to those of healthy subjects as a treatment approach, two intermediate steps have to be taken. The first step is designing and building a technically reliable system that fulfills all the technical requirements. Secondly, to iron-out all technical issues, we plan to test the system at this stage only in a healthy participant population. The current study is targeted to achieve the above two aims, by demonstrating that a subject-independent Common Spatial Pattern classifier can be used in a neurofeedback paradigm for brain pattern matching.

**Figure 1:**
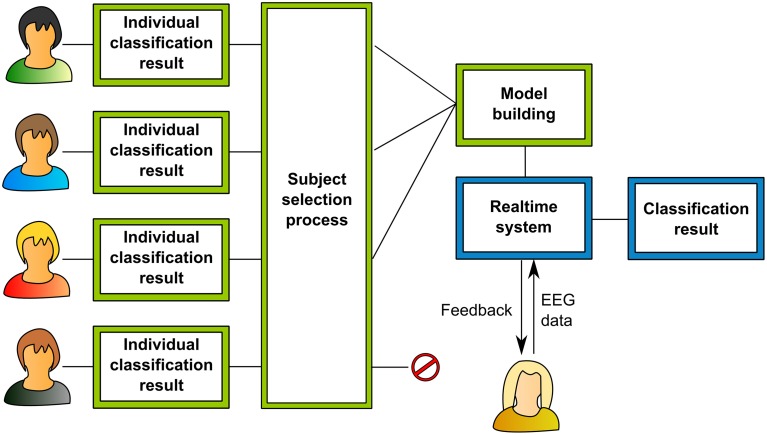
**Flow diagram explaining the two experimental stages**. The first stage is color-coded in green, the second stage is color-coded in blue. In the first stage, data is collected offline from several subjects according to the offline recording paradigm. After analysis, this data yields individual classification results for all the subjects. These results are obtained by the method shown in Figure 2. According to these results and further statistical tests the data of the most promising subjects is selected. The data of the others is rejected. A fused classification model from all the data of these subjects is created and then used in the real-time system to provide neurofeedback.

As per our aims, the current study is divided into two experimental stages (Figure 1). In the first set of experiments, a subject-independent classifier was trained from EEG data collected from a group of healthy individuals who were instructed to perform positive emotional imagery and motor imagery. The motor imagery task was chosen as a contrasting brain state to the positive emotional state in a two-class Support Vector Machine (SVM) classifier. For training the two-class SVM classifier, EEG features were obtained by spatially filtering the band-separated EEG signals by the method of CSPs (Ang et al., 2012). The CSPs of the trained classifier represented the healthy brain patterns pertaining to the two states, i.e., positive emotion and motor imagery.

Once the classifier was proven to be feasible for robust classification of positive emotion and motor imagery in different participants, a second stage experiment tested whether the classifier could decode brain states and provide feedback in real-time. Five new individuals participated in a neurofeedback experiment, in which they were trained to replicate the common brain states of the participants of the first experiment, aided by the feedback provided by the subject-independent classifier.

The result of this study shows that the classification of these brain states can be performed in real-time and used as a neurofeedback system, and demonstrates the technical feasibility of the subject-independent pattern classification approach.

## Materials and methods

### Real-time pattern classification

A real-time pattern classifier is constructed in two stages: An offline stage in which the classifier is trained on previously recorded data, and an online stage in which the trained classifier is used to provide feedback to the participant in real-time. In the offline stage, a classification model is trained by extracting relevant features from the data. These features capture the most discriminative characteristics of the different classes of data. In the online part, the model is applied to new data in real-time. The class-label that is predicted by the classifier at every time point is converted to feedback by the neurofeedback system.

**Figure 2:**
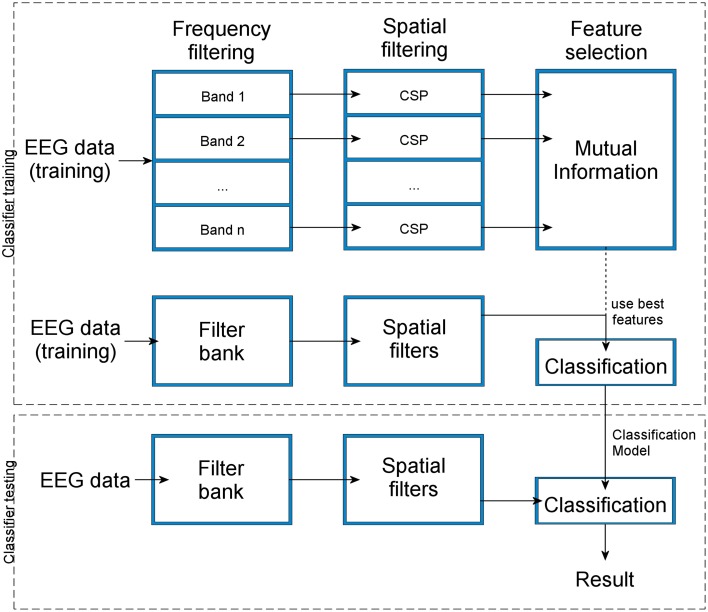
**The flow-chart shows how the consecutive steps of frequency filtering and spatial filtering of the training data and automatic feature selection lead to the construction of a classification model that can be used for classifier testing with new data**. The result of the classification is the estimation of a class label for the testing data set.

In our implementation, the classification system is built to discriminate between two classes using trial-by-trial EEG data. The approach is based on the Filter Bank Common Spatial Patterns algorithm (Ang et al., 2008, 2012; Figure 2). In the first step, a filter bank is created by repeatedly band-pass filtering the raw EEG data using Chebyshev type 2 filters. In the next step, the data in each frequency band is spatially filtered by the method of CSP. This procedure yields features for each of the bands. These features are finally evaluated by computing their mutual information with the label of the data from each trial, i.e., the class-label, and thus the most discriminative pairs of CSP and frequency bands are selected. The selected features are then used for the classification. A more detailed description of this method is given in the following sections.

#### Feature extraction

The algorithm of CSP can be understood as a method that creates weight maps of the channels of the EEG signal. The weight maps reflect the importance of the signal content of the channels for separating the conditions encoded in the data (Blankertz et al., 2008). The maps are essentially spatial filters that are projected onto data. By projection of these filters, the data is transformed to maximize the ratio of the variance of the EEG amplitudes between the two conditions. Therefore, the variance of the filtered signal can be used as a discriminative feature for a classification task.

The approach is based on the simultaneous diagonalization of two matrices as described in Fukunaga (1972). CSP has been used for feature extraction in EEG data classification for the first time in a study by Koles et al. (1990), and in several studies that followed (e.g., Müller-Gerking et al., 1999; Ang et al., 2008, 2012).

The decomposition of one trial of EEG data can be described as,

$$\begin{array}{l} Z=P^{T}E. \end{array}$$

Where *E* represents a single trial of band-pass filtered EEG time series. *Z* denotes the EEG time series *E* after spatial filtering by the CSP projection matrix *P*^*T*^. This projection matrix is computed by solving the Eigen-decomposition problem,

$$\begin{array}{l} S_{x}=U\psi_{x}U^{T};x\in \left\{a,b\right\}, \end{array}$$

where *S*_*x*_ originates from transformations of the sample covariance matrices of the trials of the two classes in the EEG data. *U* is the matrix of eigenvectors of *S*_*x*_ and ψ_*x*_ are the eigenvalues. The index *x* may be substituted by *a* and *b*, representing the labels of the two classes. A brief explanation of the transformations is given below; a formal description can be found in Fukunaga (1972). Koles and Lazar (Koles et al., 1990) describe the use of this approach in an experimental setting with EEG data.

For obtaining *S*_*x*_ the sample covariance matrix of the data of each condition is calculated, normalized with respect to its trace and averaged over trials. These matrices are combined to form a composite covariance matrix that is factored into its eigenvectors. These eigenvectors are used to formulate a whitening transformation that renders the composite covariance matrix isotropic. The same transformation is applied to the individual trial-averaged, normalized sample covariance matrices of the EEG data of each class. It has been shown that after this application the two transformed matrices share the same eigenvectors, and the sum of their eigenvalues λ is 1 (Fukunaga, 1972).

Using the eigenvectors, the projection matrix can be computed as,

$$\begin{array}{l} P=UW \end{array}$$

where *W* denotes the aforementioned whitening matrix. The first and last *m* rows of the projection matrix *P* are used to decompose the EEG into CSP.

After that the classification features *F* are extracted from the decomposed EEG time series *Z* by computing and normalizing its variances by the first *m* and last m rows of *Z*,

$$\begin{array}{l} F=\log\left(\frac{var\left(Z\right)}{\sum_{i=1}^{2m}var\left(Z\right)}\right). \end{array}$$

#### Automatic feature selection based on mutual information

After the extraction step, a set of pattern features exists for each band in the filter bank. The optimal subset of these features is selected from the whole set of features by a mutual information-based algorithm. Mutual information is an information theoretic quantity that measures the mutual dependence of two random variables (Cover and Thomas, 2006). In the training phase of our classification system, the two random variables are: (1) the variable that represents the extracted features, and (2) the variable that represents the class for every trial, for the whole duration of the EEG acquisition.

Mutual information can be formulated as:

$$\begin{array}{l} I\left(F,\omega \right)=H\left(\omega \right)-H\left(\omega |F\right) \end{array}$$

where *F* denotes the set of features, and ω denotes the class labels (Ang et al., 2012). *H*(ω) is the entropy defined as:

$$\begin{array}{l} H\left(\omega \right)=-\sum_{\omega =1}^{2}\;p\left(\omega \right)\log_{2}\;p\left(\omega \right). \end{array}$$

The conditional entropy is given by

$$\begin{array}{l} H\left(\omega |F\right)=-\sum_{\omega =1}^{2}\;p\left(\omega |F\right)\log_{2}\;p\left(\omega |F\right) \end{array}$$

Bayes rule is used to compute the conditional probability of the class given the features:

$$\begin{array}{l} p\left(\omega |F\right)=\frac{p\left(F|\omega \right)p\left(\omega \right)}{p\left(F\right)}. \end{array}$$

After computing the mutual information for all the available features and the class-labels of the corresponding data trials, they are sorted by descending value of mutual information, and the *k* best ones are selected.

#### Support vector machine

Classification is the task of estimating the class label of a given data sample on the basis of a trained model. From the data one or more characteristic features are extracted. The model for classification is built by feeding a set of labeled training features into the classifier.

A SVM is a type of classifier that maximizes the separation between two classes of data by finding a hyperplane that separates a high dimensional feature space into two subspaces of distinct classes. The estimation of the class label is carried out by computing the signum of the decision function *y*^*i*^ that is defined as,

$$\begin{array}{l} y^{i}=w^{T}x^{i}+b \end{array}$$

The hyperplane is optimal if the objective function *L* is minimized

$$\begin{array}{l} L=\frac{1}{2}w^{T}w+C\sum_{i=1}^{N}\xi^{i} \end{array}$$

under the constraint

$$\begin{array}{l} y^{i}\geq 1-\xi^{i}\text{ with }\xi^{i}\geq 0 \end{array}$$

The *N* feature vectors *x*^*i*^ are weighted by the vector *w*. Here, *b* is a constant bias value. A margin of ξ around the hyperplane is allowed to account for misclassification of each index *i* of the feature vector if a dataset cannot be separated without classification error. The slack variables ξ can be weighted by C.

The hyperplane is a linear decision boundary in a feature space that optimally separates points of one class-label from the other. It is defined by the closest feature vectors called support vectors, from which the name of the classification system SVM is derived (Schölkopf et al., 1999; Laconte et al., 2005). Once the hyperplane is found, the unknown class-label of a new feature point can be easily predicted based on its position with respect to the decision boundary.

### Experimental setup and data flow

Our algorithm is implemented in the Matlab interpreter language (Matlab Inc., Natick MA, US). It works on EEG signals in a trial-by-trial manner, i.e., the data is recorded continuously and then split into a number of trials (e.g., 64) of equal duration (e.g., 5 s) for each condition.

**Figure 3:**
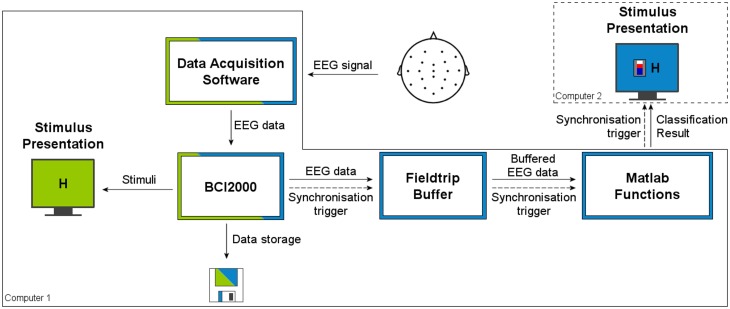
**The general setup of hardware and software used for our experiments**. The color-coding of the flow-chart indicates the experiment type. Components shown in a green color were used in the offline setup. Blue marks components that have been used in the real-time setting. Components with both colors were used in both experimental setups. All software components surrounded by a solid line were installed on the data processing computer, the ones surrounded by a dashed line were run on the computer for stimulus presentation.

The general setup of the hardware for conducting our experiments can be seen in Figure 3. The data acquisition system was connected to a computer for collecting data offline, storing it and training the classification models. This setup was expanded for the real-time experiments by adding another computer for the presentation of stimuli and neurofeedback. Thereby, data acquisition and processing and stimulus presentation could be uncoupled.

In both, the offline and real-time setups, the BCI2000 software was used to retrieve the data from the acquisition system and the Fieldtrip Buffer was used to access the buffered signal in Matlab (Schalk et al., 2004; Oostenveld et al., 2011).

The experiments for our study were conducted at the Institute for Medical Psychology and Behavioral Neurobiology at the University Clinic of Tübingen and the Escuela de Medicina, Universidad Católica, Santiago de Chile as part of a research collaboration between the two institutions. Two comparable hardware and software setups were used for our experiments depending on the institution where the experiments were conducted. The EEG signals where recorded from 28 channels and the EOG was captured with four channels. In both experimental series the signal was sampled at 500 Hz. For further information on the implementation of the classification system and the experimental setup please see the Supplementary Material, Section Methods.

### Classification parameters

For both, offline classification and real-time neurofeedback sessions we used the same parameters of the classification software. The filterbank ranged from 0 to 36 Hz and consisted of 6 bandpass frequency filters, each one with a bandwidth of 6 Hz. We chose *m* = 2 as the number of spatial filters to use for the CSP algorithm in accordance to prior studies with CSP (Müller-Gerking et al., 1999; Ang et al., 2012). The number of features to be extracted from the data was *k* = 4. We tested different values for *k* with our data, and the results showed that the average classification accuracy over all subjects did not differ significantly, but the standard deviation of the classification results was lowest when *k* = 4.

### Generation of visual feedback

**Figure 4:**
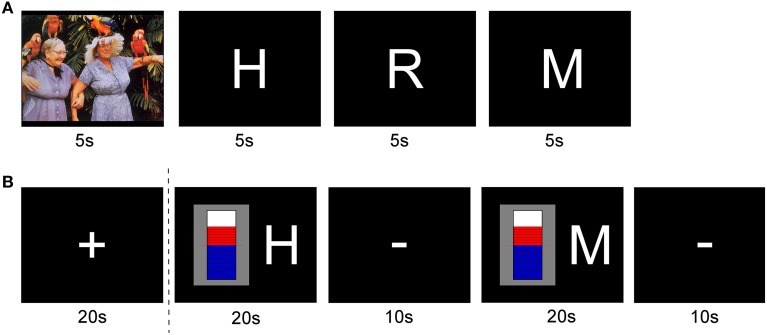
**Block design protocols for conducting offline classifier training and neurofeedback sessions**. **(A)** Experimental cues, and their onsets and durations during data acquisition for offline classification. **(B)** Experimental cues, and their onsets and durations for online classification and the visual feedback for the neurofeedback training.

Real-time feedback of the brain states was given by changing the bars of a graphical thermometer (Figure 4B, second block) in proportion to the output of the online SVM classifier. The feedback was initialized with the baseline value, which was represented by 10 blue bars that reached up to the dashed red line in the middle of the thermometer. In each update interval one bar was added to or removed from the thermometer, according to the sign of the output of the SVM, i.e., the classification result. The bars above the baseline value were colored in red. The letter besides the thermometer indicated the type of imagery (positive emotion or motor imagery).

### Bias correction of the SVM

Systemic changes, e.g., alignment of the EEG cap and slight differences in recording impedance, as well as mood and concentration level of the subject potentially introduce a bias of the classifier toward one condition (Sitaram et al., 2011). We corrected for this bias by first collecting EEG signals during a baseline period when the participant was instructed to focus on a fixation cross, and not move or perform any mental imagery. We then subtracted the mean of the SVM values during the baseline period from the SVM values in the real-time feedback.

### Experimental paradigm

The experiment was divided into two stages: In stage one, data for training the classification model was recorded and later used to test the classification system offline. In stage two, a fused classification model was built from the data of stage one. The model was used to provide neurofeedback to two new healthy subjects. In the first stage, data from 27 healthy female participants between the age of 18 and 29 (average age: 24.6) was recorded. For the first 23 subjects, EEG data was recorded with the BrainAmps system. The EEG data of the last four subjects was recorded with the NuAmps system. Each subject was seated in a comfortable chair in front of a screen for presentation of stimuli.

Ethics approval was given by the Medical Faculty of the University of Tübingen and the Medicine Department of Universidad Católica de Chile. Informed consent was obtained from all subjects before the experiment.

#### Offline classifier training

Each recording session used a block-design protocol (Figure 4A). The core components of the experimental paradigm were two types of imagery. In one condition cued by the letter “H,” participants were asked to perform mental imagery of a happy situation. In the other condition cued by the letter “M,” the task was to perform motor imagery. One session comprised five runs of 4 min each. During these 4 min, 4 different stimuli were shown repeatedly for 5 s each. A pause of 2 s was introduced between two stimuli. The order of the stimuli was deterministic. The first stimulus was a previously selected image from the International Affective Picture System (IAPS, Lang et al., 1997), the second one was the letter “H” representing the word “Happy,” followed by the letter “R” for “Rest,” and finally the letter “M” for “Motor.” The letters were presented in a size that was easily readable for the participants. They performed happy imagery during the presentation of the letter “H,” counted backwards during the letter “R,” and performed motor imagery during the letter “M.” There were eight regulation trials for each condition per run, totaling 80 regulation trials for one session.

Participants were instructed well in advance to identify several emotional episodes from their personal lives, so that they could use those episodes during happy imagery. Also, before the experiment, participants were asked to identify one image from the IAPS that best epitomized “happiness.” To remind and strengthen their emotional recall strategies, the pre-selected IAPS images were included in the block design preceding the happy imagery block, as a reminder of the specific type of emotion imagery to employ.

For the motor imagery block, subjects were instructed to perform kinesthetic motor imagery of an action involving opening and closing of both hands repeatedly (e.g., squeezing a small ball). The hand movement was shown to the participant prior to the experiment, and was practiced several times under supervision from the researcher.

#### Online classification and neurofeedback

In the second stage of the experiment, a fused classification model was built from the data of all the 16 subjects with a mean classification accuracy of 75% or greater. During preprocessing, the EEG data was normalized to a range between −1 and 1. The 1.5% largest and the 1.5% smallest values in the EEG signal were identified and considered as outliers and replaced by the new maximum or minimum value of the normalized range, 1 or −1. Our signal showed that, on subject average, amplitude values of about −100 and 100 μV lay above the 1.5% percentile and below the 98.5% percentile. The amplitudes of the EEG for waking adults usually lie between 10 and 100 μV (Niedermeyer and Silva, 2005). The normalization was necessary to even out absolute differences of the values in the data between subjects and the two different acquisition systems.

The data from the 16 subjects was then concatenated and a classification model was created according to the aforementioned methodology. The total number of trials used for creating the model was 1280 (80 ∗ 16).

For the feedback training runs, we recruited five healthy subjects who had not been part of the first set of experiments. The new subjects were instructed to learn to control the feedback signal (Instruction: “Make the feedback thermometer move up!”). They were notified that the feedback signal comes from a classifier that contains information from other subjects' brain states, and that the matching of their own brain activity/state with the classifier information would allow them to control the feedback signal. As the original subjects performed happy and motor imagery, the new subjects were also instructed that it might be easier to match the original brain states performing those mental actions (cued by the letter H and M in the experimental paradigm).

The real-time feedback paradigm consisted of the following blocks (Figure 4B): In the very beginning of each session a “+” sign was shown (fixation period). Subjects were instructed to fixate on the plus sign, and to avoid moving or blinking. In the first regulation block, the letter “H” for the happy imagery was shown right next to the feedback thermometer. The second regulation showed the letter “M,” indicating the motor imagery. Between each of these blocks, the sign “−” was presented to indicate a resting period. During this period, subjects were allowed to blink, relax and get ready for the next task block. After the first four out of the eight regulation blocks an additional fixation period “+” was shown. Thus, the classification system could readjust the bias of the SVM for the second half of the run. Each of the blocks was presented for 20 s, except for the resting periods which lasted 10 s. A whole real-time testing run lasted 5 min. The update interval for the feedback was 1 s. The data that was used to classify and adjust the feedback accordingly comprised the data of the last second. According to the sampling frequency of 500 Hz, 500 data points were used.

Data from the subjects was measured in three sessions comprising four runs each. Therefore, the number of regulation trials per session was 32 and the total number of regulation trials was 96. The three sessions were conducted on three different days across 2 weeks.

## Results

### Offline-classification results

In order to analyze and assess the quality of the data of the 27 participants, we performed visual inspection of the EEG signals in the Brainvision Analyzer software (Brain Products GmbH, Gilching, Germany). EEG signals from all subjects were of good quality, and hence no part of the data was rejected.

**Figure 5:**
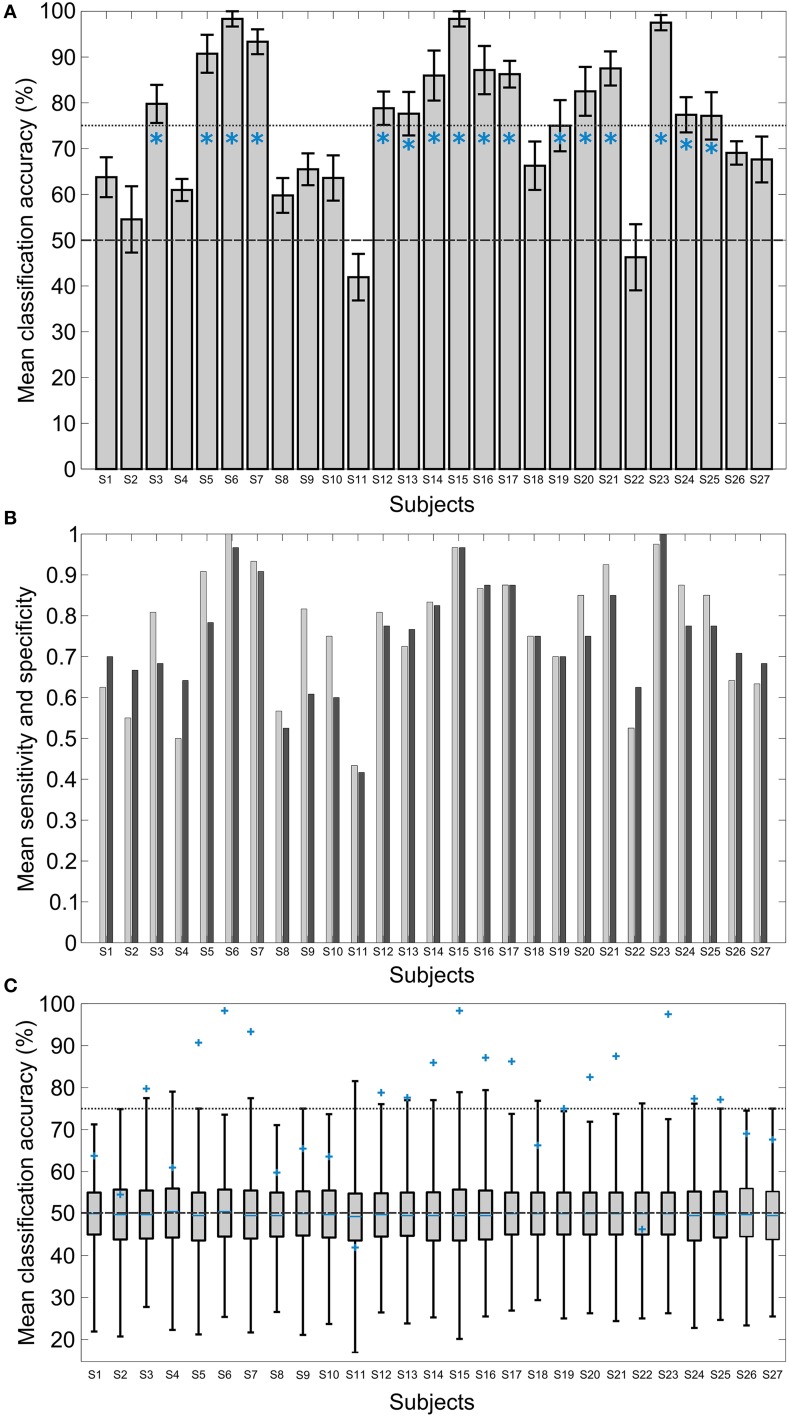
**Results of the offline classification for all the subjects in a 10-times 10-fold cross-validation test and the 1001 repetitions of a randomization test**. **(A)** Mean classification accuracies in percent, i.e., the number of correctly estimated class labels in the test set. The mean level of 50% is marked by a dashed gray line. The standard deviation of the 10 repetitions are shown with black indicators. The dotted gray line marks the 75% level. The blue asterisks indicate the subjects that were chosen for the subject-independent classification model. **(B)** Sensitivity (light gray) and specificity (dark gray) of the classifier, averaged over all permutations of the cross-validation test for the individual subjects. **(C)** Boxplots of the results of the randomization test. The boxes show the first and the third quartile of the classification accuracies of the randomly labeled data. The short blue lines show the median. 99.9% of the data lie within the bounds indicated by the whiskers. The blue markers report the results of the cross-validation with correctly labeled data. The dashed gray line marks the 50% mean accuracy level and the dotted gray line the 75% mean accuracy level.

Figure 5A shows a bar graph of the classification accuracies of all 27 participants in a 10 times 10-fold cross-validation test: In this test the 80 raw EEG trials were randomly permuted for each participant. In 10 repetitions, 90% of the trials were used for feature extraction and classifier training, and the remaining 10% were used for classifier testing. This procedure was repeated 10 times for the data of each participant. The trials were randomly permuted to generate a new composition of the folds in each repetition. The classification accuracies indicate the number of times that the classifier predicted the label of the testing trials correctly, averaged over all the permutations. The standard deviation of the accuracy of the 10 repetitions of the cross-validation was within the bounds shown by black lines above and below each of the bars. The average classification accuracy for all the cross-validation runs of all the participants was 75.30%. For Subjects 11 and 22, the classification accuracy is below chance, indicating that the classifier was not able to devise a good model. Figure 5B shows sensitivity and specificity of the classifier for the cross-validation test. To ascertain the statistical significance of the results of the offline classification experiment, a randomization test was carried out. In this test, for each subject 1001 repetitions of the 10-times 10-fold classification with our system were executed. The labels of the trials in the data were randomly permuted in each repetition. The results of this test showed that the individual classification accuracy of these subjects with correctly labeled data lie outside of the 99.9% percentile of the results of the randomization test (Figure 5C).

In order to assess the validity and robustness of the fused model, another cross-validation analysis, with 20 folds repeated 10 times, was performed with the concatenated data from the 16 chosen subjects. The classification accuracy averaged over the 10 repetitions was 61.5% with a standard deviation close to zero.

**Figure 6:**
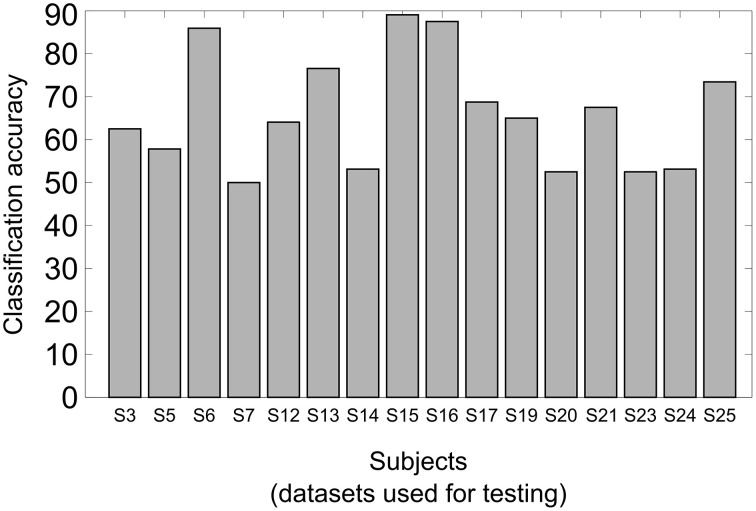
**Results of the leave-one-subject-out analysis**. Sixteen different models have been built training the classifier on the data of 15 subjects and testing the classifier on the data by the remaining subject, indicated on the x axis. The classification accuracies are shown on the y axis.

Furthermore, a leave-one-subject-out-analysis has been carried out with the data of the selected subjects. Sixteen models have been trained on the data of 15 subjects and tested on the data of the remaining one. The bar graph in Figure 6 shows the classification accuracies of the analysis when the data was tested on the subject indicated on the x-axis, i.e., the data of this subject had no influence on the model. The classification accuracy averaged over all the 16 tests was 66.2% with a standard deviation of 13.2.

**Figure 7:**
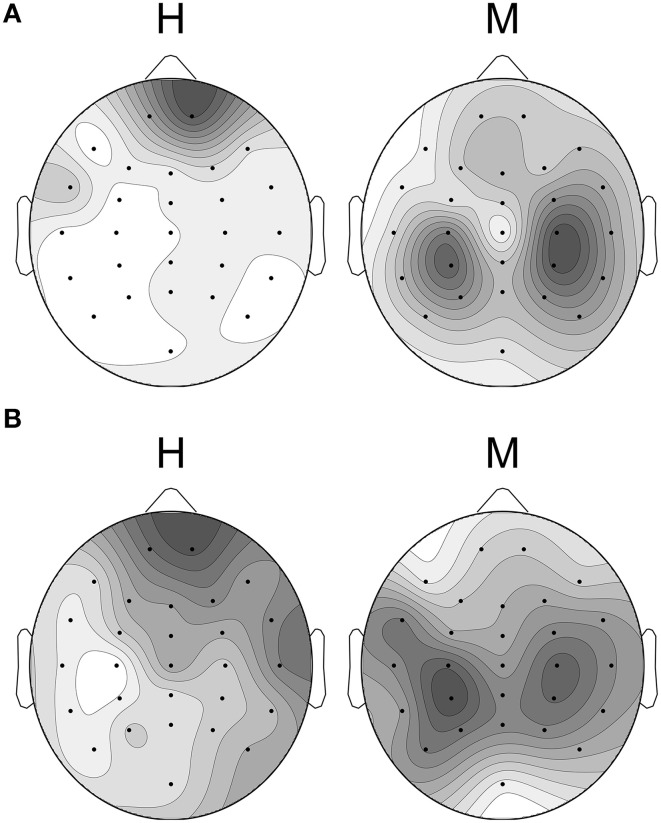
**The most prominent Common Spatial Patterns (CSPs) extracted from the classification model and from a combination of subjects**. Black dots mark EEG channels. The darker the gray value in the plot is, the more prominent is the ratio of variance in the respective channels. The topographic map on the left shows the pattern for the happy condition, whereas the pattern of the motor condition is shown on the right. **(A)** The CSPs extracted from the concatenated data of the 16 well-performing subjects were used to create the classification model. **(B)** Individual CSPs averaged over all the subjects with a mean classification accuracy of 75% or more. Black dots mark EEG channels.

Figure 7A shows the CSP extracted from the fused model used in the neurofeedback sessions. The left topographic plot represents the pattern for the “happy” condition, and the right one shows the pattern for the “motor” condition. The black dots indicate EEG channels. These patterns show the channels of the EEG that contain the most discriminating information for the classification of the two brain states. The darker the gray value is in the map, the more prominent is the ratio of variance in the corresponding channel. Therefore, these patterns can be interpreted as weight-maps indicating the contribution of the individual channels toward classification. The left topographic map shows greater involvement of the frontal cortex in the “happy” as compared to the “motor” condition. The right plot, on the other hand, indicates that the data from the channels over the motor cortex were more important to classify this condition. Figure 7B shows the most prominent CSP, i.e., the first and last column of the inverse projection matrix *P*, averaged over all the subjects with a classification accuracy of 75% or greater (*n* = 16). To generate the plots, for each subject, the absolute values of the CSPs of each cross-validation run were averaged. Then, the values of these composite individual CSPs were scaled to a range of 0–1 to ensure that the data of each subject contributes equally to the final averaged plot.

The classifier selected bands two and three of the filter bank for feature extraction, i.e., the band from 6 to 12 Hz and from 12 to 18 Hz.

### Online-classification and neurofeedback results

**Figure 8:**
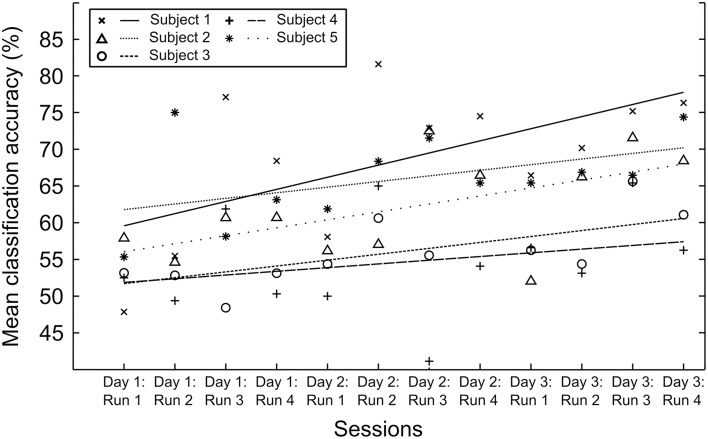
**Classification accuracies of the real-time neurofeedback sessions on three consecutive days for the five healthy subjects**. Fifty percent is the random level. The markers indicate the mean classification accuracy of all the regulation trials of the corresponding run. The lines are trend lines, computed from the mean classification accuracy. Run 4 on day 2 had to be excluded from the data of Subject 3 because the subject reported concentration issues due to external disturbance.

The results of the real-time feedback sessions are presented in Figure 8. The first healthy subject showed an increase of the classification accuracy of 62.2% averaged over the four runs of the first session on day 1 to 71.6% averaged over the four runs of the session of the second day. One week later, in the third session, the results were still stable with an average classification accuracy of 72.0%. The second subject achieved an average classification accuracy of 58.5% on the first day. On the second day the average classification accuracy increased to 63.0% and on the third day to 68.0%. The third and fourth subjects started with a classification accuracy close to chance. Their classification accuracies increased to 61.9 and 57.41%, respectively, due to subsequent training. The fifth subject was able to increase his/her average classification accuracy from 61.8% on the first day to 70.2% after the final training session.

The above values were computed by counting how many labels were estimated correctly by the classifier. As the feedback interval was 1 s, there were 152 data samples in each run for which the classifier estimated the class labels.

Sensitivity and specificity of the classifier are shown in the Supplementary Figure 1.

For further investigation of potential learning effects, we attempted to quantify the similarity of the CSP of all subjects throughout the course of the neurofeedback training. The CSPs for each run of each subject were extracted from the EEG data recorded during the sessions. The two most prominent CSP vectors were combined and their similarity with the CSPs extracted from the fused classification model were computed. The methods used for comparing the CSPs were: correlation coefficient, mutual information and Euclidean distance. All three methods produced similar results, and hence for conciseness we will only present the results of the correlation method.

**Figure 9:**
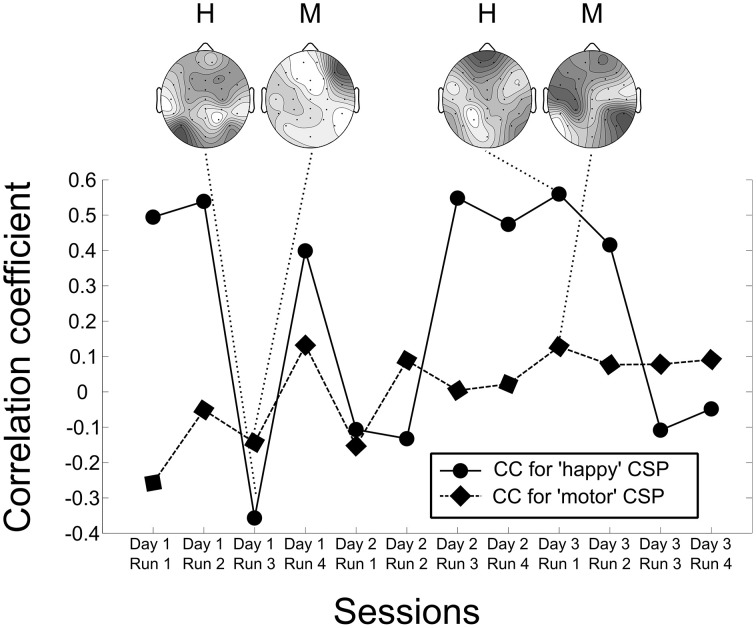
**Dynamics of the two most prominent Common Spatial Patterns over the course of the experiment for the first subject**. The figure plots the similarity index, i.e., the correlation coefficient of the subject's CSP of a particular run and the CSP of the model, against the number of runs. Four topographical plots show the CSPs for data of runs with the lowest and the highest similarity values for visual comparison with the classification model seen in Figure 7A. The topographical plots marked by “H” represent the pattern for the happy condition, the ones marked by “M” the pattern for the motor condition.

Figure 9 shows the dynamics of the two most prominent CSP over the course of the experiment for the first subject. The figure plots the similarity index against the number of runs. Two topographical plots show the CSPs for data of runs with the lowest and the highest similarity values.

Linear regression models between the similarity indices and the classification accuracies were created for all subjects. Table 1 shows the *r*^2^-values of the regression, giving an estimate of how well the similarity of the pattern predicts the classification accuracy.

**Table 1:** **The correlation coefficient (CC) between the subject's Common Spatial Patterns in all the runs and the CSPs of the classification model has been computed**.

**Subject**	**CC: happy condition**	**CC: motor condition**
Subject 1	0.0138	0.0182
Subject 2	0.00654	0.0553
Subject 3	0.00169	0.274
Subject 4	0.00091	0.0148
Subject 5	0.0114	0.000535

*Regression analysis has been carried out to quantify the relationship between the similarity value (CC) and the classification accuracy for all the runs of the individual subjects. The r^2^-values are shown here. With one exception, the motor condition in Subject 3, they are all very low, indicating no (linear) relationship between the similarity index and the classification accuracy*.

## Discussion

The aim of the present study was to demonstrate a new method for real-time subject-independent pattern classification and neurofeedback of brain states from EEG signals and to assess the feasibility of this approach as a precursor to its application in a planned, future study on patients with difficulties achieving a healthy affective state (e.g., depressive disorders).

The present implementation of the classification system is based on the application of spatial filters on EEG data in different frequency bands (Ang et al., 2008). Spatial filtering is achieved by the method of CSP and the best features, i.e., the most discriminative pairs of CSPs and frequency bands are selected by a Mutual Information-based approach (Ang et al., 2012). The procedure was applied and tested in an offline experiment with 27 healthy subjects and subsequently in a series of sessions with five subjects who received real-time neurofeedback.

The result of the offline experiment with a mean classification accuracy of 75.30% for the 27 subjects shows that the classification system at hand can reliably decode the two types of imagery, i.e., emotional imagery and motor imagery. The spatial patterns yielded by the algorithm and averaged over subjects indicate that the channels in the frontal regions are important for discrimination of the happy imagery condition, whereas for the motor imagery condition the channels above the central brain areas are important. The findings for the motor condition are in line with previous studies on classification of motor imagery (Müller-Gerking et al., 1999). The importance of frontal regions for the emotional imagery condition is concordant with the extensive literature signaling the involvement of frontal areas in emotion processing and regulation (Phan et al., 2002; Ochsner et al., 2004; Kohn et al., 2014).

To choose the subjects to include in the fused classification model incremental training and testing of the model with different thresholds of the individual mean classification accuracy (i.e., 60, 65, 75, 80, 90%) were carried out. In each iteration subjects with an individual mean classification accuracy of above the threshold value were in included. A threshold value of 75% was the best choice for having enough number of training samples for generalization, i.e., all trials from 16 subjects, while maintaining good data quality, which means sufficient information in the data for reliable classification. The classification results for these subjects with the correctly labeled data lay outside of the 99.9% percentile of the distribution of the classification test with randomly permuted class labels, which shows that the classification results are very significant and suited to be used for the second stage of the experiment.

The CSP plot that was generated for Figure 7B shows very similar patterns to the one that we extracted from the subject-independent classification model (Figure 7A). In addition to the online classification results, this shows that removing outliers and normalizing and concatenating the EEG data before applying the classification algorithm is a viable method for creating the classification model.

Additional offline analysis was carried out to ascertain the performance of the classifier. Although, the subject-independent offline cross-validation analysis showed a relatively low classification accuracy of 61.5%, the low standard deviation of the mean classification accuracies during the cross-validation test indicate stable performance.

Besides, we have also included the data from the “Rest” trials in another round of analyses. The classification system was able to distinguish “Happy” vs. “Rest” trials and also “Rest” vs. “Motor” trials with an average accuracy of 69.5 and 78%, respectively (Please see Section Classification analysis Including the “Rest” Trials of the Supplementary Material).

The leave-one-subject-out-analysis showed performance comparable to the subject-independent offline cross-validation analysis. The average classification accuracy for 11 subjects lay well above chance, for some even close to 90%. However, for five subjects, namely S7, S14, S20, S23, and S24, the system was not able to achieve above chance accuracy. Subjects S7 and S23 were among the best in the cross-validation analysis carried out for the individual subjects. One interpretation of this result might be that these subjects exhibited a very clear and distinct brain activation pattern that was easily classifiable individually but that was quite different from that of the other subjects to be included in a fused classifier. The individual classification accuracies and their high statistical significance in terms of the randomization test with permuted class labels may neither be the only nor the best criterion for the selection of subjects for the fused classification model. A method that selects subjects according to comparison of their individual CSP could be devised. However, such a method has pitfalls of its own that need to be addressed, as we explain in Section Online-classification and Neurofeedback Results and in the following paragraph.

Another possibility to find the best fused model from our data would be to run a large series of tests that builds many of such models derived from subgroups of our population of healthy subjects and tests and validates them against the remaining data. However, this kind of “brute-force” analysis requires substantial computing power and time, a potential topic of a future investigation.

The online experiment in the five new subjects demonstrates that mental states can be decoded from brain activity in real-time to provide neurofeedback. The results from the three sessions of Subjects 1, 2, and 5, which were distributed across 2 weeks, show a reliable classification accuracy that increases in the beginning and becomes stable in the last session. The upwards trends for these subjects shown in Figure 8 indicate a learning effect of the training with the classifier. Subjects 3 and 4 were not able to improve the classification accuracy throughout the course of the training to the level of the other subjects. Nonetheless, the absolute increase on average for Subject 3 is 10% points, which is a considerable increment. The results of Subject 4 show a general upwards trend. However, this increase is not significant. The reasons for that could be that some subjects need longer to find good strategies to match the modeled brain patterns and some are simply not able to perform the necessary imagery in a consistent and persistent manner. In spite of the general upward trend the observation can be made that the classification accuracies are not increasing monotonically over time. This fact can be attributed to session-to-session variability in the internal state of the subject, i.e., levels of concentration, fatigue and motivation but also to trying different imagery strategies. As one of the external factors, noise from the EEG may corrupt the data. Both lead to variations in the performance of the classifier. Furthermore, there is numerous prior evidence that learning to control brain signals by neurofeedback is not necessarily a monotonically increasing process (Gruzelier et al., 1999; Subramanian et al., 2011; Linden et al., 2012).

The block length for the imagery trials has been increased from 5 s in the offline case to 20 s in the online case as suggested in the literature (Ruiz et al., 2013b; Sulzer et al., 2013).

The results of the investigation of the dynamics of the individual CSPs of each subject do not show a clear positive correlation of the similarity indices and the classification accuracy. For all of them the goodness-of-fit of the linear regression model is low. There could be several reasons for this to happen. Although computing the similarity measures with linearly increasing random noise showed the expected result of almost linear decrease of the similarity indices (see Supplementary Figure 2), these measures might still not be optimal to quantify similarity of CSPs in a useful way as they firstly might not fully capture the information conveyed in the patterns and secondly might not be able to account for variations in the patterns, e.g., slight spatial shifts.

Furthermore, it is important to understand that the CSP show the regions that exhibit the most discriminative information of the data used to compute them. In the optimal case, “pattern matching” could be measured by comparing the CSPs extracted from the data during the real-time experiments to the ones from the model because the patterns extracted from the subjects' data and the patterns of the model would be very similar. However, under the conditions of real-time experiments this might be an overoptimistic expectation. There are factors that influence the data and, therefore, change the subject's CSPs. Consider the occurrence of movement artifacts, for example. Especially when only looking at a few data samples the respective CSPs might be influenced by artifacts that exhibit high variances (Blankertz et al., 2008). The classifier, however, going a step further by extracting (weighted) features from the EEG transformed by the filter matrices of the fused model, could still prevail and extract the relevant information, as it relies on the transformation matrix of the model and its weights learned in prior. For these reasons the expectation that a high classification accuracy inevitably leads to an increased similarity of the subjects' CSPs to the model CSP cannot always be fulfilled.

Moreover, CSP show regions of high information content for the discrimination of two conditions (Müller-Gerking et al., 1999). That means that patterns for one condition change when the brain-state of the other condition changes. As an example, let us assume that during a run of neurofeedback the subject is able to exhibit the desired brain activations for one of the conditions. For the other condition, however, she is exploring a new mental strategy. This would lead to high classification accuracies at least for the first condition because the classifier is extracting features based on the group model. The CSPs, on the other hand, may look completely different to the ones of the model, even though the classifier can classify the brain state successfully.

Considering the above, we propose that the classification accuracy is a better measure than the similarity of CSP for assessing the performance and the learning effect of the subjects. Our results from the online training showed that new subjects are able to “match” the brain patterns of a fused classifier based on the brain signals of a different group of individuals, aided by the feedback provided by the subject-independent classifier. Future experiments could investigate if prolonged training leads to further increase and more stable classification accuracies and patterns. More robust and meaningful similarity measures for CSP could also be investigated.

Our results have important implications for future experiments on BCIs and its potential clinical applications. For example, for depressive disorders, in which current treatments are commonly based on antidepressants and/or psychotherapy (Lam et al., 2009; Parikh et al., 2009; Patten et al., 2009; Gelenberg et al., 2010), several attempts have been made using neurofeedback. However, the majority of previous neurofeedback studies have attempted to train patients to achieve a healthy brain state aided by the feedback of their own brain activity. As an example, the most used protocols of EEG based neurofeedback in depression have focused on Alpha band (and its inter-hemispheric asymmetry) and Theta/Beta ratio within the left prefrontal cortex (Choi et al., 2011; Dias and van Deusen, 2011; Escolano et al., 2014) in an effort to correct abnormal patterns of brain electrical signals. These systems have been built upon the idea that a patient can learn by practice to consciously generate healthy brain states. However, patients could have difficulties finding healthy patterns of brain activity. A subject-independent classifier, which provides neurofeedback information of a healthy pattern of brain states, could offer a novel alternative for patients suffering from brain disorders characterized by an abnormal mood or affect (e.g., depression).

Furthermore, many neurofeedback studies have provided feedback of neural information coming from a few sources or circumscribed brain areas (for example: Ruiz et al., 2013b; Sitaram et al., 2014; Young et al., 2014). The pattern classification system allows the feedback of distributed patterns of activity of the brain, accounting for the coordinated action of multiple networks, such as schizophrenia (e.g., Gaspar et al., 2009; Fitzsimmons et al., 2013; Ruiz et al., 2013a, 2014a) and autistic disorders (e.g., Just et al., 2012; Maximo et al., 2014).

Besides that, different patients might have different brain alterations although sharing the same clinical diagnosis. Hence, the use of a particular brain pattern or signal coming from the patients' brain activity for neurofeedback might not be appropriate for all patients. A subject-independent classifier that offers the patient a healthy brain state to “match” by neurofeedback, can offer an interesting alternative for both the problem of the heterogeneity of brain abnormalities among patients, and the involvement of distributed brain regions in neuropsychiatric disorders. Future studies should explore in detail the clinical benefits of this new approach.

### Conflict of interest statement

The authors declare that the research was conducted in the absence of any commercial or financial relationships that could be construed as a potential conflict of interest.
