# Supplementary material for: A subject-independent pattern-based Brain-Computer Interface
Source: Front Behav Neurosci. 2015 Oct 20;9:269. doi: 10.3389/fnbeh.2015.00269 (PMC4611064; doi:10.3389/fnbeh.2015.00269)
Supplement: Supplementary file 1 [file SupplementaryData.DOCX]

Supplementary Material

A subject-independent pattern-based Brain-Computer Interface

Andreas M. Ray^1†^, Ranganatha Sitaram^1,2†^*, Mohit Rana^1,2,3^, Emanuele Pasqualotto^4^, Korhan Buyukturkoglu^1^, Cuntai Guan^5^, Kai-Keng Ang^5^, Cristián Tejos^6^, Francisco Zamorano^7,8^, Francisco Aboitiz^9^, Niels Birbaumer ^1,10^, Sergio Ruiz ^1,9^ *

*** Correspondence:**

Sergio Ruiz
Departamento de Psiquiatría
Centro Interdisciplinario de Neurociencia
Escuela de Medicina
Pontificia Universidad Católica de Chile
Street Marcoleta 391, 2 Piso
32349 Santiago, Chile
E-mail: [smruiz@med.puc.cl](mailto:smruiz@med.puc.cl)

Ranganatha Sitaram, ME, PhD
Institute of Medical and Biological Engineering, School of Engineering
Section of Neuroscience and Dept. of Psychiatry, School of Medicine
Pontificia Universidad Catolica de Chile, Santiago, Chile

# Methods

**1.1. Implementation of the classification system**

Our algorithm is implemented in the Matlab interpreter language (Matlab Inc., Natick MA, US). It works on EEG signals in a trial-by-trial manner, i.e. the data is recorded continuously and then split into a number of trials (e.g. 64) of equal duration (e.g. 5s) for each condition. The latency, duration and the label identifying the condition of the trials are specified by the experimental paradigm. The composition of the filterbank, i.e. the number of bands and their bandwidth, the number of Common Spatial Patterns to be used for classification $m$, and the number of features $k$ that the automatic feature selection algorithm chooses can be set by the user.

The offline implementation of our system comprises capabilities for testing the classification model by cross-validation, and thereby retrieving estimates of the classification accuracy, sensitivity and specificity of the classifier. Additionally, randomization tests can be carried out for assessing the statistical significance of the results of the cross-validation.

For online classification, the continuous data coming from the EEG amplifier is buffered until a certain number of data samples, e.g. 500, have accumulated. This package of data samples is spatially filtered by the filter bank derived from the best CSPs during the model training. The spatially filtered data is fed into the classifier, which then predicts the class-label of the EEG trial (Figure 1).

**1.2. Experimental setup and data flow**

The general setup of the hardware for conducting our experiments was as follows (Please see figure 3 of the main text). The data acquisition system was connected to a computer for collecting data offline, storing it and training the classification models. This setup was expanded for the real-time experiments by adding another computer for the presentation of stimuli and neurofeedback. Thereby, data acquisition and processing and stimulus presentation could be uncoupled. In the offline setting a second screen was connected to the computer for stimulus presentation. In the real-time setting this screen was connected to the stimulus computer. The two computers in this setup were connected via LAN and by a R232 serial connection. The LAN connection was used for sending data packets containing the classification results, and the serial connection was used for sending timing triggers for synchronizing both computers with the acquisition system. All the computers used in the experiments were state of the art workstations with modern operating systems and hardware with sufficient computational resources for real-time data processing.

In both, the offline and real-time setups, the BCI2000 software was used to retrieve the data from the acquisition system (Schalk et al., 2004). In the offline case, BCI2000 was also used to present the stimuli. The algorithm, implemented in Matlab, was used after recording to analyze the data and to train a classification model. For support-vector classification we used the LibSVM library for Matlab (Chang and Lin, 2011). In the online case, several software packages had to be used to ensure timely processing of the data. The real-time implementation of our algorithm used the Fieldtrip Buffer to access buffered packages of data coming from BCI2000. The Fieldtrip Buffer is part of the Fieldtrip Toolbox (Oostenveld et al., 2011). BCI2000 sends a trigger signal with a specified interval to Matlab. As soon as one of these triggers reaches Matlab the system computed the classification estimate of a label for the current data in the buffer. This result and the synchronization trigger are sent to the stimulus computer. Here, the Presentation® Software by Neurobehavioral Systems, Inc. ([www.neurobs.com](http://www.neurobs.com)) is in control of the timings for the stimuli and their presentation. The software waits for the synchronization trigger and the classification result and adjusts the contents of the stimulus screen accordingly.

The experiments for our study were conducted at the Institute for Medical Psychology and Behavioral Neurobiology at the University Clinic of Tübingen and the Escuela de Medicina, Universidad Católica, Santiago de Chile as part of a research collaboration between the two institutions. Two comparable hardware and software setups were used for our experiments depending on the institution where the experiments were conducted. The main exception was the data acquisition software that had to be adjusted to the acquisition system. In Tübingen, the BrainAmps 32-channel EEG system was used (Brain Products GmbH, Gilching, Germany). 28 of these channels were used to capture the EEG data. The accompanying proprietary software was BrainVision Recorder. At Universidad Católica, the neuroscience lab uses the Nuamps 40 channel EEG acquisition system by Compumedics Neuroscan with the company’s Acquire software (Compumedics Limited, Abbotsford, Australia). Again, the EEG signals where recorded from 28 channels and the EOG was captured with four channels. In both experimental series the signal was sampled at 500 Hz.

The data was also recorded in BCI2000 and stored to disk at the same time for further offline analysis. The SVM values, the bias and the values of thermometer feedback were stored on disk for every second to evaluate the results after the experiments.

# Results

**
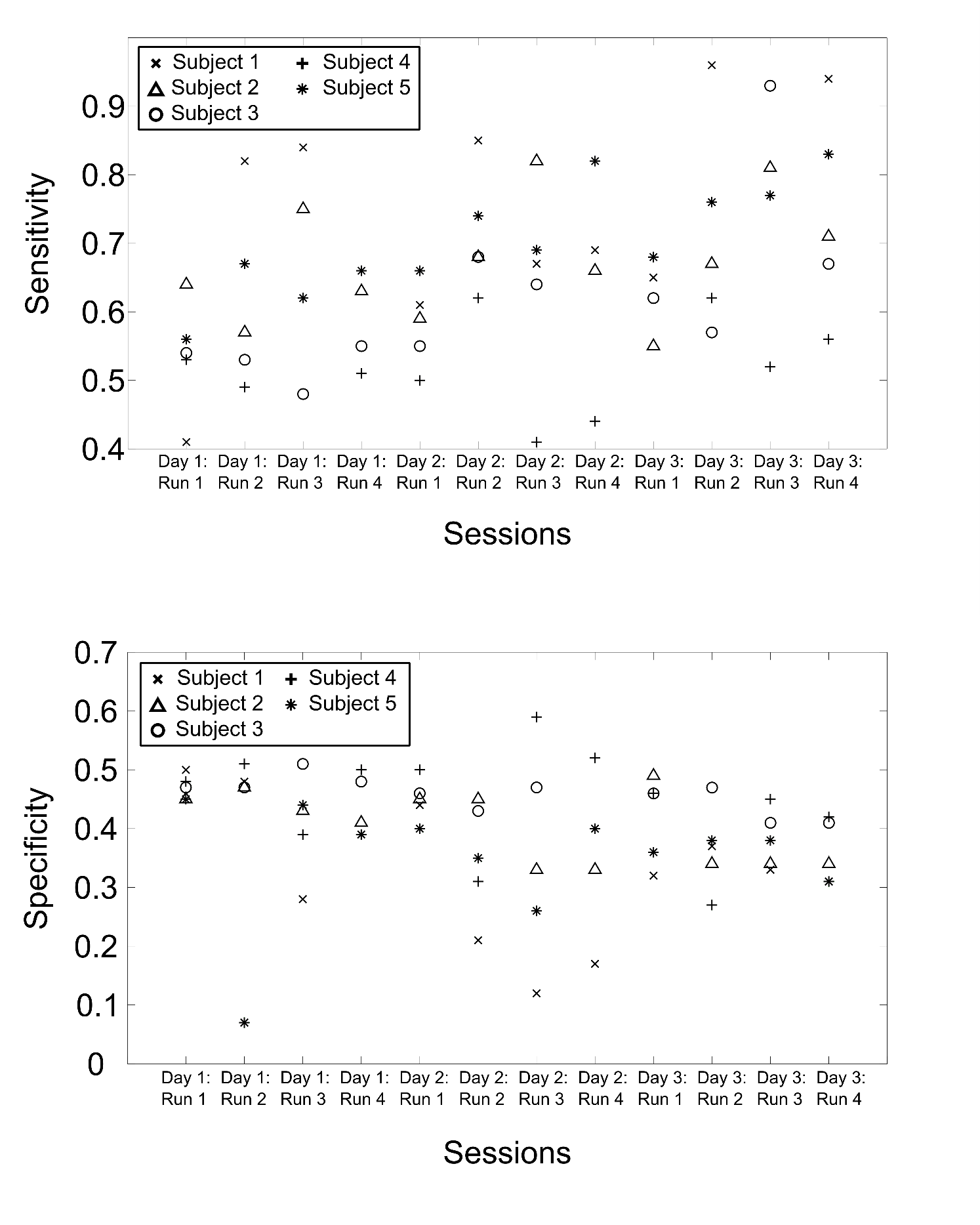
**

**Supplementary Figure 1**: Sensitivity and specificity of the online classification system. We have interpreted the classification task as a detection problem where the system has to detect the “happy” condition. That means that detection of the “happy” pattern during the H-blocks stands for true positives, whereas detection of the “motor” pattern during the M-blocks stands for true negatives. The sensitivity is generally high and increasing, whereas the specificity is relatively low. Run 4 on day 2 had to be excluded from the data of subject 3 because she reported concentration issues due to external disturbance.

# Discussion

## The correlation coefficient as measure for similarity of CSPs


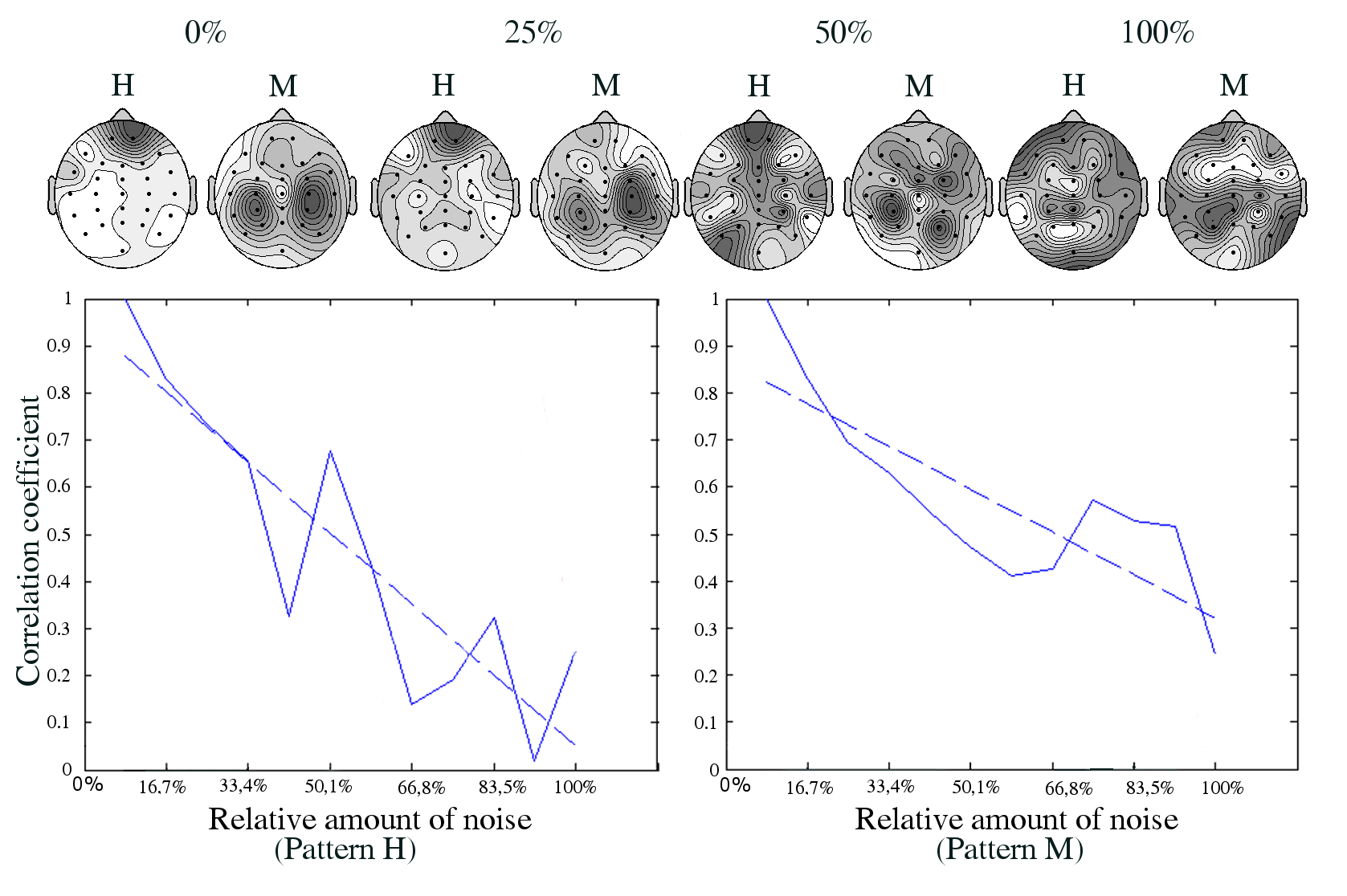


**Supplementary Figure 2**: Random noise has been gradually added to the model Common Spatial Patterns (top left: no noise) to test the correlation coefficient as a method to quantify similarity between CSPs. The line plots show how CSPs with different amounts of noise are correlated to the model CSP for the happy pattern (left line plot) and the motor pattern (right line plot). Above, the two patterns are shown with increasing amounts of noise (from left to right). The dashed trendlines represent a good linear fit.

## Classification analysis including the ‘Rest’ trials

The plots where created classifying the ‚Happy‘ vs. the ‚Rest‘ trials in the data of 11 subjects and the ‚Motor‘ vs. ‚Rest‘ trials in 8 subjects (Supplementary Figures 3-6). The other subjects had to be excluded because there where too few ‚Rest‘ trials that were not contaminated with eyeblink artifacts.

The plots indicate that a robust differentiation between Happy (H) and Rest (R) is possible for our data. Furthermore, Motor (M) seems to be distinguishable from Rest. From the results we show in the manuscript we know that the Motor and Happy (H) are also distinguishable. The three patterns appear similar: Prefrontal activation is shown for the happy condition in H vs. M as well as in H vs. R. The similarity of the M around C3 and C4 in both comparisons is not neglectable. The activation patterns for ‚Rest‘ seem to be concentrated in the parietal/occipital regions. With these results in mind, we state that we are able to extract three distinct patterns for the three different conditions.


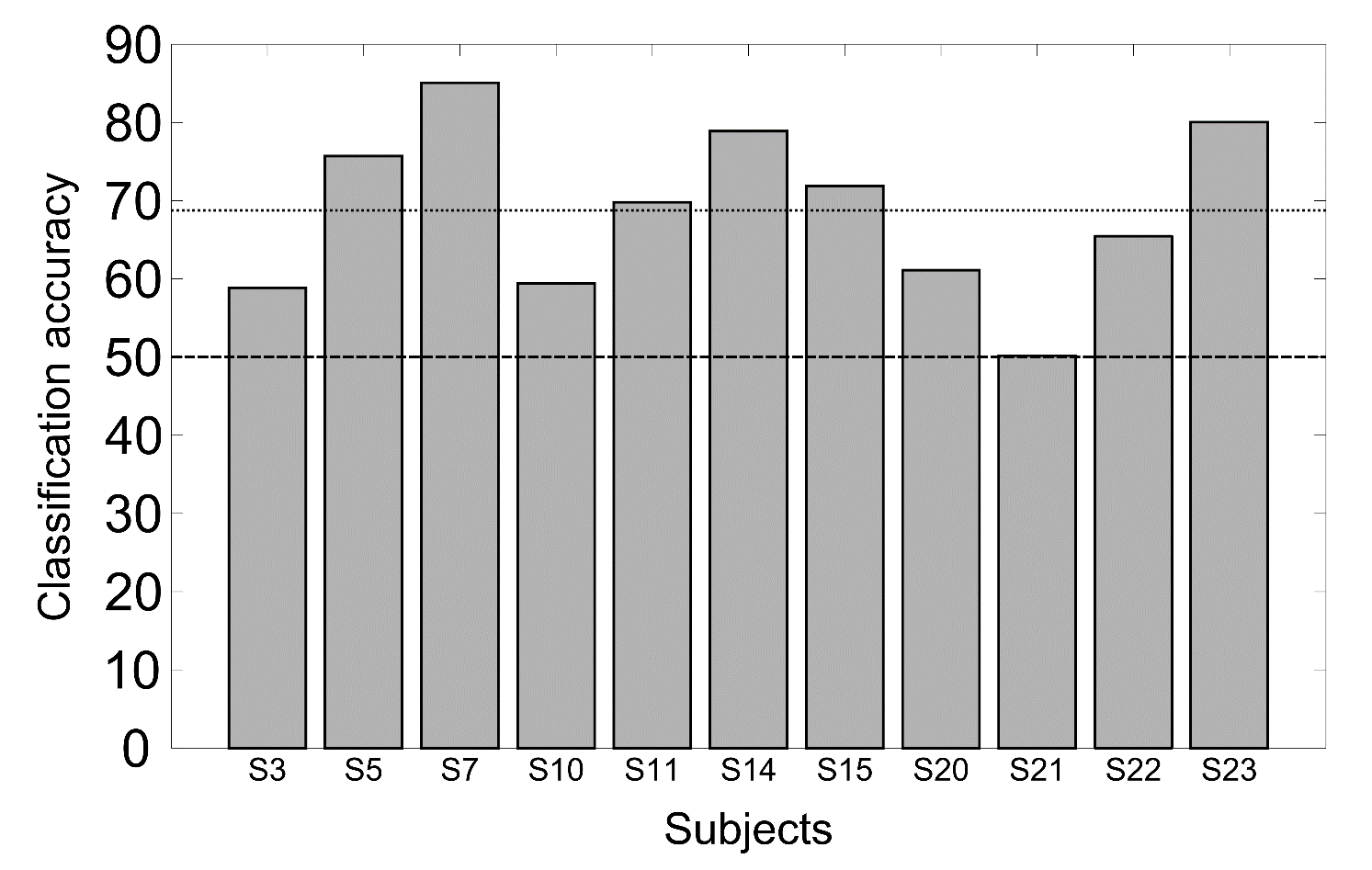


**Supplementary Figure 3**: Classification accuracies of the ‘Happy’ vs. ‘Rest’ condition (12x8 fold cross validated) in eleven subjects. Average classification accuracy across the subjects is 69.5%.


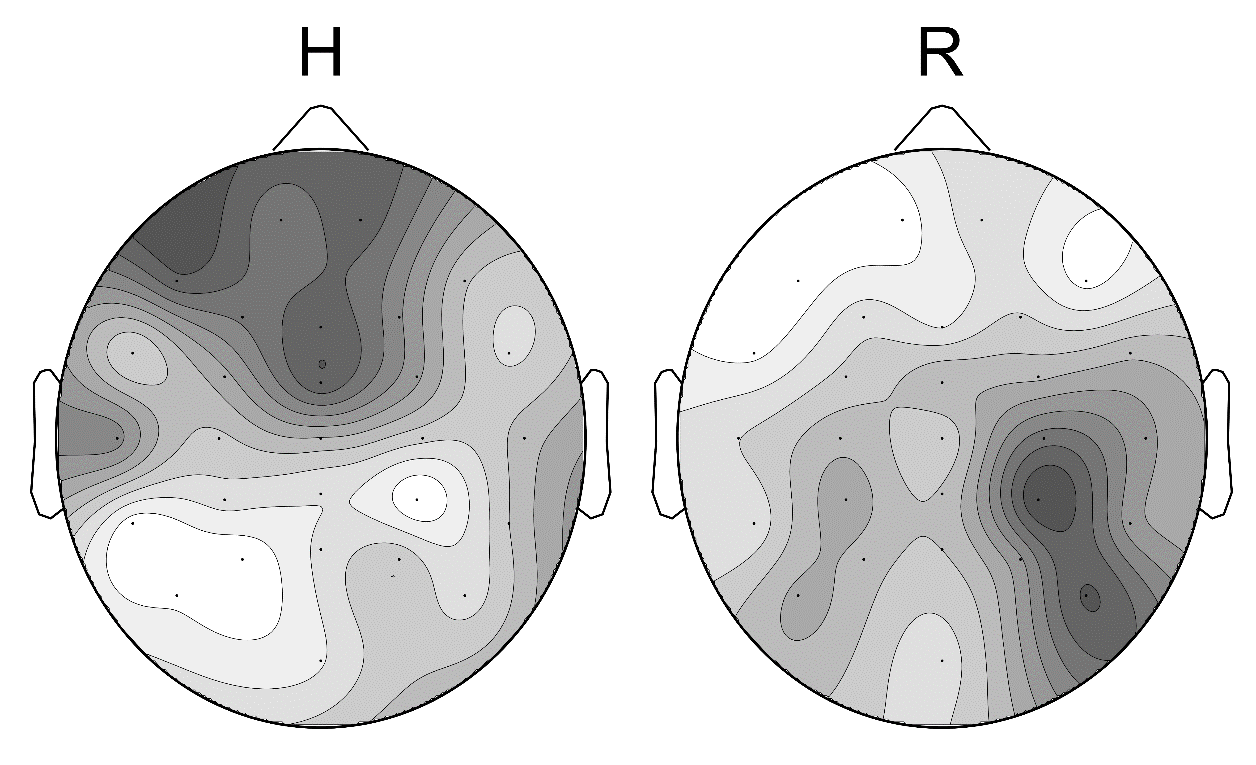


**Supplementary Figure 4**: CSP plot for ‘Happy’ vs. ‘Rest’ for the above eleven subjects, averaged across subjects.


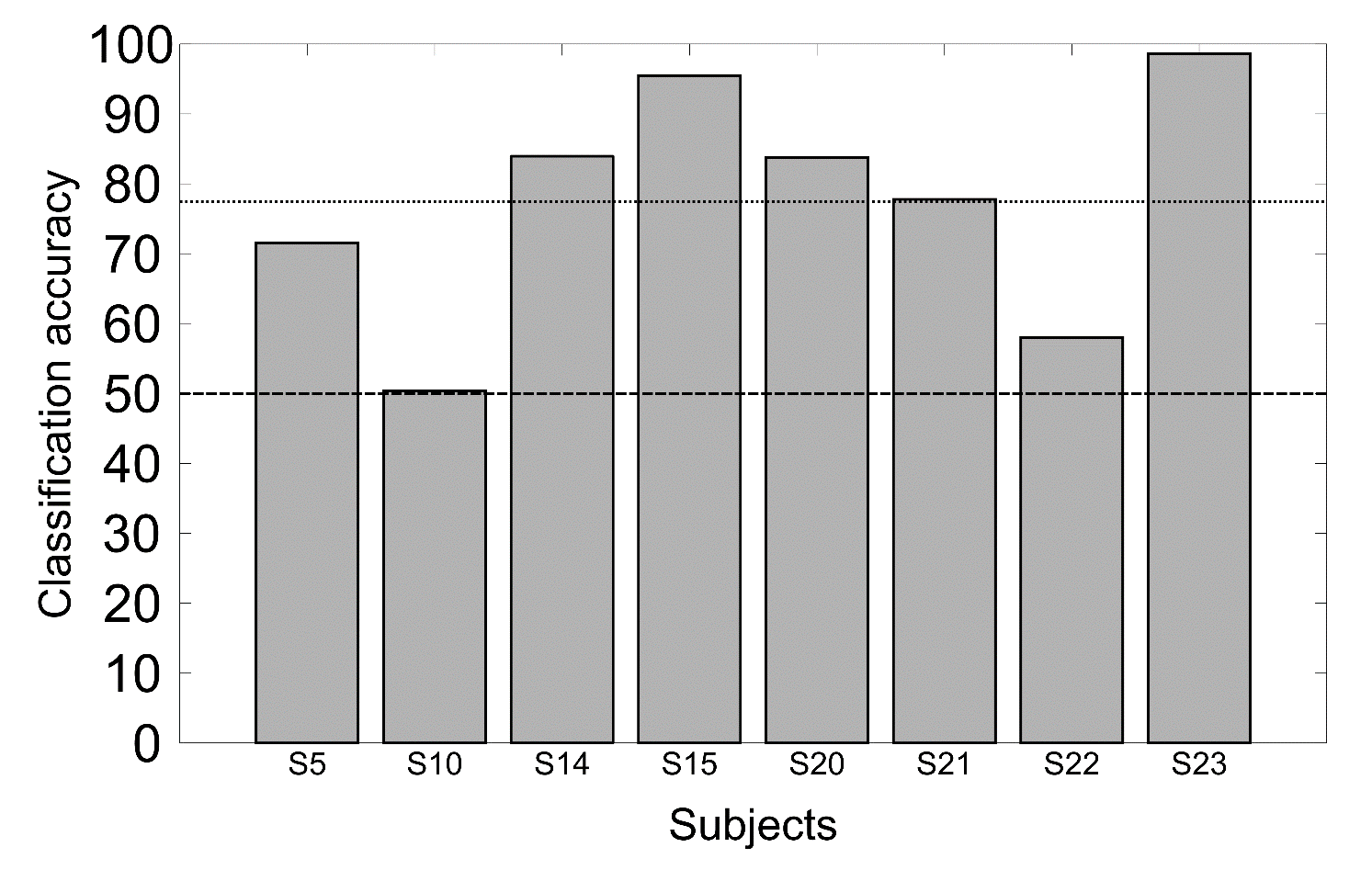


**Supplementary Figure 5**: Classification accuracies of the ‘Rest’ vs. ‘Motor’ condition (12x8 fold cross validated) in eight subjects. Average classification accuracy across the subjects is 78%.


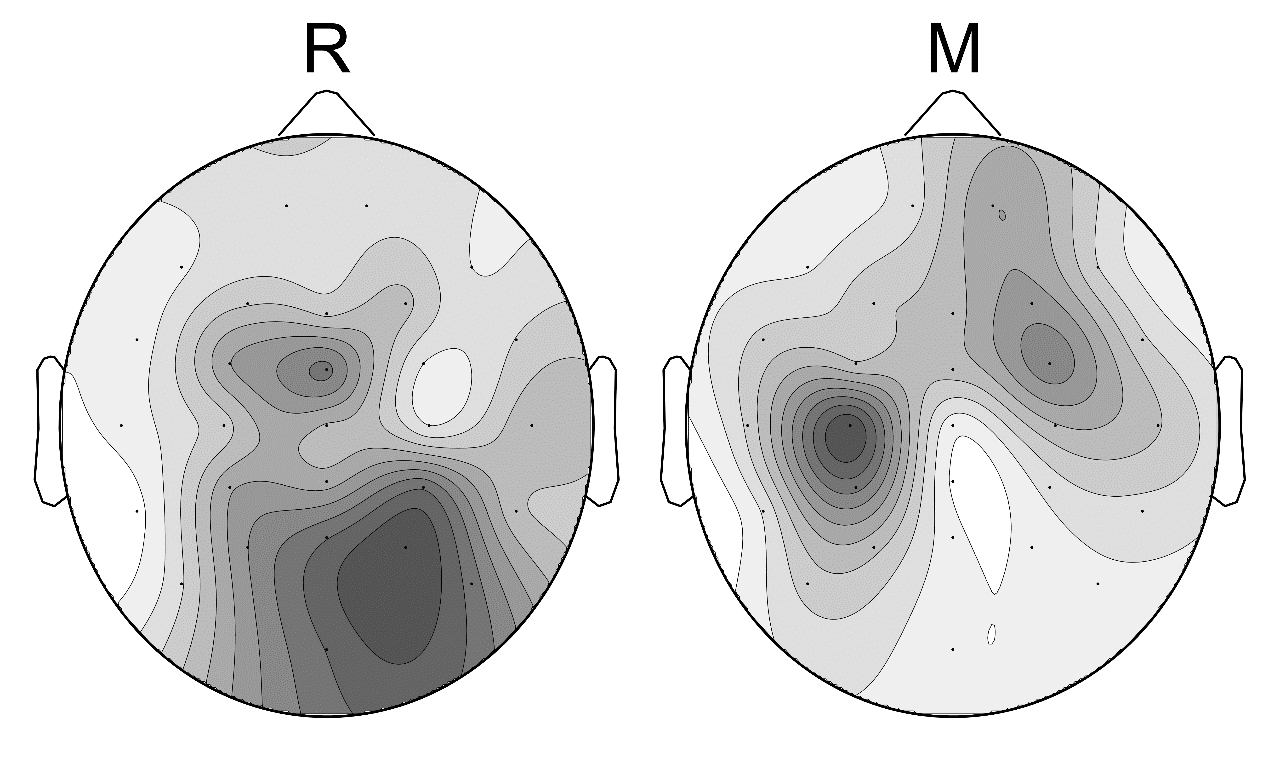


**Supplementary Figure 6**: CSP plot for ‘Rest’ vs. ‘Motor’ for the above eight subjects, averaged across subjects.
